# Difficulties in Practice

**Published:** 1869-07

**Authors:** 


### DIFFICULTIES IN PRACTICE.

Many and various are the difficulties encountered by the skillful and observing Dental operator in the management and treatment of the natural teeth. A case in illustration of this statement was this day presented.

A young man, about twenty years of age, had ten or twelve teeth filled about fifteen months ago; three or four of the fillings were large, the remainder of medium size ; some in the sides, others in the masticating surfaces. The dentine round nearly all these fillings were defective, decay to a greater or less extent having taken place ; in some just about the margins of the fillings—in others penetrating to considerable depth. The fillings gave evidence of having been well introduced and consolidated, and the supposition is that the adaptation had been good ; nevertheless, it is possible that there may have been defects in this respect. Upon examination and the preparation of other cavities, the enamel and dentine were both found to be very friable, easily disintegrated, so that it was almost impossible to make a smooth, even border to the cavity, and correspondingly difficult to make a perfect adaptation of the filling to such surface ; and in such cases there is very great liability of comminuting or breaking the dentine about the orifice of the cavity, or the angle constituted by the wall of the cavity and the surface of the tooth about it. Such injury is very liable to be done by forcing the plugger against the dentine at the point in question, or even with the burnisher if the angle is not well protected by the gold. Indeed, in some instances, is the dentine so frail that even driving the gold against it will produce the injury, though the instrument does not touch the dentine.

In all cases where a disintegration is thus effected, however well the cavity may be filled in other respects, decay will very soon occur.

Such teeth are certainly very unfavorable for successful operations, even in the hands of the most skillful—much more so in the hands of the unskillful.

The question occurs at this point: What is the best method of procedure?

In the first place, let there be no abrupt over-jutting portions of enamel or dentine about the orifice of a cavity to be filled; let the walls of the cavity be as nearly parallel with each other as possible all the way from the orifice to the bottom. In the next place remove the angle of enamel or dentine at the orifice, either with a reamer, which makes instead of the angle a concave bevel, and leaves two obtuse angles instead of one acute or right angle; a better plan is, with the proper cutting instrument and file, to remove the angle and substitute the round or bead form to the edge of the orifice; the size of this should be governed by the condition of the dentine.

Another particular requiring close attention is the introduction, adaptation and consolidation of the filling, especially at the orifice. Perhaps the most thorough adaptation of the filling upon such a border can be obtained by the use of non-adhesive gold foil; and this condensed with the most finely serrated pluggers, those of such form and cut and used in such a manner that the instrument will in no case touch the dentine, and let the gold be built up till every point of the border that has been cut by the chisel or file shall be perfectly protected. Care and skill are required in such cases in the use of the burnisher, lest by its heavy contact the enamel is broken. The round or bead like form of the angle at the orifice is not perhaps, in all cases, the best. For instance, in cavities upon the crown surfaces of the molars, in the depressions, when the cusps of opposing teeth strike, and the surface of the filling is necessarily to be concave; but in all cases of friable teeth, when the surface of a filling can be convex, the form suggested is preferable. T.

